# Eyes on the road: brain computer interfaces and cognitive distraction in traffic

**DOI:** 10.3389/fnrgo.2023.1171910

**Published:** 2023-05-26

**Authors:** Victoria Bosch, Giulio Mecacci

**Affiliations:** ^1^Machine Learning, Institute of Cognitive Science, Osnabrück University, Osnabrück, Germany; ^2^Department of Artificial Intelligence, Donders Institute for Brain, Cognition and Behavior, Radboud University, Nijmegen, Netherlands

**Keywords:** BCI (Brain Computer Interface), human-machine interaction, neurotechnology, traffic, societal impact of AI, ethics of AI, cognitive distraction

## Abstract

Novel wearable neurotechnology is able to provide insight into its wearer's cognitive processes and offers ways to change or enhance their capacities. Moreover, it offers the promise of hands-free device control. These brain-computer interfaces are likely to become an everyday technology in the near future, due to their increasing accessibility and affordability. We, therefore, must anticipate their impact, not only on society and individuals broadly but also more specifically on sectors such as traffic and transport. In an economy where attention is increasingly becoming a scarce good, these innovations may present both opportunities and challenges for daily activities that require focus, such as driving and cycling. Here, we argue that their development carries a dual risk. Firstly, BCI-based devices may match or further increase the intensity of cognitive human-technology interaction over the current hands-free communication devices which, despite being widely accepted, are well-known for introducing a significant amount of cognitive load and distraction. Secondly, BCI-based devices will be typically harder than hands-free devices to both visually detect (e.g., how can law enforcement check when these extremely small and well-integrated devices are used?) and restrain in their use (e.g., how do we prevent users from using such neurotechnologies without breaching personal integrity and privacy?). Their use in traffic should be anticipated by researchers, engineers, and policymakers, in order to ensure the safety of all road users.

## 1. Introduction

Recent developments in neuroscience and engineering are making brain-computer interfaces (BCIs) increasingly accessible and inexpensive for use outside of medical applications. Brain-computer interfaces allow indirect measurement of the neural activity of the user. Medical applications range from predicting the onset of epileptic attacks and speaking assistance for patients with locked-in syndrome. Outside the medical field, and progressively entering the consumer market, we see professional as well as recreational applications, examples being mediation guidance, social media and hands-free gaming (Hayden, 2020; Snap, 2022; Macrotellect, n.d.; Muse, n.d.; Neuralink, n.d.). The ability of BCIs to enable hands-free typing would make it possible to send text messages or search for information on the web, and this novel way of navigating the digital world may have large implications for how we navigate the physical world. Non-motoric, hands-free device control makes BCIs particularly interesting in the context of travel and vehicle operation in traffic, where they could be used to mentally operate vehicle interfaces like GPS or driving support (Louveton et al., 2017; Chavarriaga et al., 2018; Nissan, 2018; Mercedes-Benz Group, 2021; Yang and Van Hulle, 2023), or for non-travel related applications like mobile communication such as writing messages or initiating phone calls. Because we acknowledge that, for the aforementioned reasons, these devices might now or in the near future raise commercial interest, we would like to draw attention toward potential misconceptions or undesirable implications at the earliest possible stage of development and deployment. It may be suspected that due to reduced visuomotor interference, BCI-based hands-free devices may be less distracting than other voice-controlled hands-free devices. However, we argue that they may require at least similar or higher cognitive interaction than voice-controlled applications and thus pose a serious cognitive distraction. Additionally, the distracted user may not show any behavioral signs of use, thereby hindering effective strategies of social control and containment over this technology. Effective regulation faces particular challenges regarding the integrity and privacy of the user. We argue that more attention must be devoted to the possible inclusion of BCIs in the daily lives of people in the near future. As BCIs may become more popular and could be worn throughout the day, we must expect and anticipate the novel ways in which humans will guide their attention and behavior, as well as how they navigate the world. Therefore, analysis and quantification of potentially novel cognitive distractions in traffic, and the inclusion of wearable neurotechnology in the legal framework, are necessary and highly recommendable.

Before proceeding, we would like to address a possible criticism common in the ethics of technology. Ethicists are sometimes accused (and quite commonly by other ethicists) of artificially inflating a problem or, even worse, “concern-trolling.” We have seen that happening in neuroethics within the Deep Brain Stimulation debate (Erler, 2021) but it can affect literally any discussion developing on hypothetical grounds, like artificial superintelligence (even one of the authors himself indulged in a mild, yet similar criticism in the past; see Haselager and Mecacci, 2020). The mere fact that the neurotechnology application we discuss in this paper is not (yet) commercially available or used in the relevant contexts, does not mean a preemptive discussion is not necessary or important, at least to avoid incurring in what has been defined as the “delay fallacy.” This consists in underestimating the risk until enough information about a certain technology is available (Hansson, 2004). Falling into such a trap may result in trying to address a potential problem only once it is too late to do so (Collingridge, 1980). Rather, an informed and realistic ethical discussion on technologies that have yet to come may have a significant impact in both steering technical development and informing political decision-making.

## 2. Brain-computer interfaces and traffic: potential uses and impact

Here, we will explore the potential risks associated with the use of BCIs in traffic as well as the challenges that regulation faces, starting the section with an overview of different types of commercially viable neurotechnologies.

### 2.1. Types of wearable neurotechnology: passive and active BCIs

Researchers have developed a multitude of techniques and devices to measure neural activity, of which some are likely to be used in commercial BCI devices. Among these, electroencephalography (EEG) has emerged as one of the most commonly used non-invasive brain-reading techniques and is one of the key contenders in the market of wearable neurotechnology. Although measuring only the activity of assemblies of neurons near the cortical surface, it is able to pick up signals on a sub-second timescale that are informative of key mental processes, such as attention and motor activity, and is both inexpensive and transportable (Rashid et al., 2020). Another contender for wearable BCI devices is near-infrared spectroscopy (NIRS) which, like EEG, is non-invasive and primarily measures cortical activity. Due to a lower temporal resolution, it may however not be usable for applications that need to detect neural changes on a sub-second timescale to function (Fazli et al., 2012). Invasive devices, such as subcortical implantable electrode arrays, may in a further future become viable for commercial use. A non-cranial contender for BCI wearables is electromyography (EMG), which can measure even a millimeter of muscular movement. Although technically not a BCI, it is still relevant to consider as it indirectly interfaces with the brain and can be considered to be a similar type of wearable technology that is used to control a device without significant and visible muscular efforts. An example of a wrist-based neural interface using EMG for spelling may be commercially released by Meta in the near future (Tech@Meta, 2020, 2021).

To characterize types of brain-computer interfaces in the context of traffic use, we identify an important conceptual separation between *passive* and *active* BCIs (Zander et al., 2010). Passive BCIs simply monitor neural activity and lend themselves well for monitoring the unintentional cognitive or affective state of the user, in particular in detecting driver fatigue to subvert its risks. Since a significant amount of car crashes happen due to fatigue and drowsiness (up to 50%, SWOV, 2019; European Commission, 2021), the use of passive BCIs for fatigue and drowsiness detection could be beneficial in increasing road safety, in particular for professional transport drivers (Zhang et al., 2017; Chavarriaga et al., 2018; Hwang et al., 2021; Martínez Beltrán et al., 2022). This points to the potential that these passive devices may have for the future of traffic, since common automatic fatigue detection systems, which e.g., note driving style changes, still suffer from inconsistent and invalid detection (SWOV, 2019). In order not to throw out the baby with the bathwater, we will not consider passive BCIs in the concerns voiced in this article.

Active BCIs, in contrast, use intentionally generated neural signals to control another device, such as a mobile phone. A primary example is a “speller,” with which the user can type text simply by thinking about the words or spelling them mentally. Active BCIs could also be used to hands-free control in-vehicle systems like GPS, or personal devices. These systems may be used in combination with other devices like augmented reality glasses (Hayden, 2020; Tech@Meta, 2020; Snap, 2022) or simply digital screens which can be viewed and neurally controlled.

### 2.2. Active BCIs pose increased cognitive distraction

The use of electronic devices in traffic is discouraged and regulated because they pose a serious distraction to drivers and significantly contribute to the number of accidents (WHO, 2018). Sources of distraction include manual interference and visual competition, but also interference with cognitive information processing (Strayer et al., 2015). The latter source of distraction is challenging to target explicitly, because it cannot be assessed behaviorally. However, the occurrence of external cognitive distractions can be measured and actively minimized. Active BCIs, as we will argue, may cause increased cognitive load and distraction. Their use and potential abuse should therefore be considered in the production of present and future traffic regulations.

By reducing visual and motoric interference by devices that guide the driver's gaze away from the road, one might suspect that mind-controlled, hands-free devices are an extraordinary technological opportunity to reduce distraction-induced accidents. However, several works show that the elimination of visual and motoric interference does not does not fully offset the cognitive interference that a distractor may cause, and that novel hands-free devices present unique challenges of their own. Indeed, Strayer et al. (2015) show that despite reducing visual-manual interaction, hands-free devices for calling and device operation continue to pose a significant danger due to the high cognitive workload demands. In particular, the authors show that writing emails using speech-to-text technology is cognitively more demanding than regular conversations or hands-free calling, and that this higher cognitive workload causes longer brake reaction times. While other works find that the Google Glass™ head-mounted AR-device improves upon manual cellphone texting by reducing off-road gazes in some respects, it is also found that its use still significantly impairs reaction times compared to undistracted driving (Strayer et al., 2015; Tippey et al., 2017). This is due to the cognitive distraction that the secondary task poses, which remains regardless of motoric load. In short, the cognitive load cause by a secondary task can be disentangled from the visuomotor inference caused by the particular technology that is used (Sawyer et al., 2014). Therefore, we conclude that typing text or controlling a device using an active BCI causes a comparable cognitive workload to other hands-free technology, even if all visuomotor distractions are reduced. This thus does not comply with the intuition that BCIs offer a much safer alternative to manual or voice-based hands-free device control.

Moreover, BCIs may come with their own particular challenges to the cognitive load of the driver, which can be anticipated. Tactile, auditory, or potentially even neural alerts from the wearable may be harder to ignore than a ringing phone. Indeed, it has been found that users respond quicker to wearable devices than to their mobile phones (He et al., 2018). Since the effort and speed with which the secondary task can be initiated may be high, users may multitask more than with a hand (or voice)-operated device, diminishing the potential benefits (He et al., 2018). Moreover, the proper functioning of BCIs may require even more heightened attention than other devices, as they are often developed for settings wherein the user has full concentration and no distractors (Zhao et al., 2018). Improper functioning of the BCI under multiple task demands can increase errors and stress, and thus further divert focus from the primary task at hand; operating the vehicle (Emami and Chau, 2020).

The use and marketing of active BCIs in traffic may be accompanied by the conviction that it may increase the ease of vehicle control, reducing visual-manual distractions (Louveton et al., 2017; Mironov et al., 2021). However, we argue that in the best case, those devices might not constitute a significantly less distracting alternative to simpler hands-free devices due to remaining cognitive interference, and in the worst case may be more distracting and tiring for the user. Our argument is grounded primarily in research on other wearables, and is thus indirect; we have observe a lacuna in the literature regarding the quantification of cognitive load when operating a BCI device as a distractor to a primary task. To the best of our knowledge, this has not been conducted yet, but is highly recommended to further validate or reject our concerns.

### 2.3. Regulatory challenges concerning the use of active BCIs in traffic

Active BCI-controlled hands-free devices are not explicitly included in policies concerning the use of electronic devices in traffic, while their use, as argued above, may significantly increase cognitive load and pose a distraction from driving while their use is visually undetectable. In many countries, the law on the use of mobile devices in transportation vehicles states that drivers are no longer allowed to hold an electronic mobile device used for communication or information processing while driving (WHO, 2018). However, hands-free operation of a device is often still allowed, as is operation of navigation devices, and many modern cars contain smart screens that are attached to the dashboard and can thus be used while driving. We argue that if the regulation of BCI devices in traffic is to be implemented to effectively mitigate safety problems, we are faced with several challenges that need to be addressed.

First, there is a semantic problem: when does one “use” or “hold” a device? For example, wearing a smartwatch is not thought of as holding a device, although the device can be viewed and operated similarly to a handheld phone. In the case of BCIs, they are non-motoric in nature but their use and the extent to which they cause distraction may be similar to handheld or other motoric controls, and may also make use of visual feedback. This points to an issue relating to regulation, which must find metrics, other than whether a device causes visual-motoric competition, to determine the bounds of safe secondary cognitive load.

Second, there is a detection problem: currently, observing if someone is holding or operating their device while driving is quite straightforward. Law enforcement officers can with relative ease visually assess a situation and proceed to further investigation, i.e., inviting the driver to pull off, where needed. Moreover, detecting usage is not only important to law enforcement, but also for road users to assess and predict the driving behavior of the BCI user and make safety judgements. As commercial wearable BCIs become smaller, visual assessment may become harder and harder. In combination with other interfaces such as augmented reality glasses, we can expect people to be in intense cognitive interaction with technology without showing obvious behavioral signs. Where hands-free calling or speech-to-text technology already presents a detection problem, as the only visible behavioral sign is the user talking, an active BCI user might show no visible signs whatsoever of their intense mental engagement.

Third, and last, we have to consider how intimately connected to a user a BCI system could become. Regulating these technologies might require law enforcement to violate a user's personal space to simply search for the presence of a device, which will be increasingly aggravated when using implanted technologies. Monitoring by law enforcement may, given the above-mentioned detection problem, lead to physical or digital breaches of the user's privacy. This may ultimately lead to the violation of fundamental rights (Mecacci and Haselager, 2019).

Although there has been growing interest in expanding the policies to generally prohibit the operation of devices in several countries, this is generally not yet the case, because it would also exclude the operation of navigation devices and similar relevant tasks. To our knowledge, BCIs are nowhere explicitly included in traffic legislation yet. Given their potential upcoming use, and their peculiar features, we find it necessary to raise awareness of the novel possibilities that those may introduce. The problems we stated above can only be addressed through timely technical and institutional design solutions.

## 3. Moving forward

In this article, we argue that the use of active brain-computer interfaces in traffic may potentially be harmful, and thus warrants consideration not only by researchers and engineers, but policymakers too. The use of active BCIs by road users is likely to result in levels of cognitive distraction that are comparable to or worse than other hands-free alternatives, and the regulation of their use in traffic may prove to be challenging.

The impact of the inclusion of these devices in the daily lives of people is challenging to predict. Therefore, it is recommended to research both the benefits and disadvantages of the use of BCIs in traffic and keep an open mind toward technological innovation in the coming decade(s). Since the initial use of BCIs is primarily in the medical domain as an aid for those with a disability, it is important to nuance the warning and not stigmatize the use of active BCIs. In the context of road use, these devices have the potential to greatly benefit individuals with disabilities and help them regain their independence (Audi, 2019; Cruz et al., 2021; Ping et al., 2021). It is important to recognize and harness the potential of (active) BCIs without stigmatizing or uncritically hindering their development and deployment. Rather, it is our duty as academic scholars to stimulate responsible innovation in its broadest sense, providing the right conceptual tools and ethical assessments to facilitate discussion across the widest possible range of relevant stakeholders.

One step to avoid throwing the baby out with the bathwater would be for future research to look into quantifications of cognitive distraction and accurately compare different ways of operating various devices with BCIs, voice, and other modalities. In particular, we observe a gap in the literature; while we find resources that present distractors while operating the BCI is the primary task, it is necessary to quantify mental load in paradigms wherein multitasking the BCI operation is the distractor from another primary task as well. This way, more exact tradeoffs can be assessed. Stimulating companies that develop BCIs to take traffic safety into account, the devices they develop could for example be equipped with a “driver mode” (akin to “airplane mode”) that motivates the driver to disable the active BCI, whilst simultaneously allowing the passive BCI to measure and guide their attention and alert them when they become distracted or drowsy.

As we anticipate the growing use of brain-computer interfaces in commercial settings for e.g., cognitive enhancement, communication, and gaming, we also expect their usage in everyday life and the context of traffic. Contrary to the intuition that BCIs may offer a less distracting alternative to other devices due to reduced visuomotor distraction, our analysis indicates that the use of active BCIs while driving may pose a similar or worse cognitive distraction compared to other hands-free devices. This is particularly concerning because the user may not display any behavioral signs of distraction, making it challenging to observe and regulate. As of yet, we find there is no adequate quantification of how BCIs and hands-free devices compare in this setting. While BCIs have considerable positive applications, it is crucial to evaluate their impact on the attention economy and transportation. Doing so could prevent accidents and promote the responsible and mindful use of neurotechnology.

## Data availability statement

The original contributions presented in the study are included in the article/supplementary material, further inquiries can be directed to the corresponding author.

## Author contributions

VB wrote the manuscript with input from GM. Both authors contributed to manuscript revision, read, and approved the submitted version.
